# Physical constraints on the establishment of intracellular spatial gradients in bacteria

**DOI:** 10.1186/2046-1682-5-17

**Published:** 2012-08-29

**Authors:** Carolina Tropini, Naveed Rabbani, Kerwyn Casey Huang

**Affiliations:** 1 Biophysics Program, Stanford University, Stanford, CA, USA; 2 Department of Bioengineering, Stanford University, Stanford, CA, USA

## Abstract

**Background:**

Bacteria dynamically regulate their intricate intracellular organization involving proteins that facilitate cell division, motility, and numerous other processes. Consistent with this sophisticated organization, bacteria are able to create asymmetries and spatial gradients of proteins by localizing signaling pathway components. We use mathematical modeling to investigate the biochemical and physical constraints on the generation of intracellular gradients by the asymmetric localization of a source and a sink.

**Results:**

We present a systematic computational analysis of the effects of other regulatory mechanisms, such as synthesis, degradation, saturation, and cell growth. We also demonstrate that gradients can be established in a variety of bacterial morphologies such as rods, crescents, spheres, branched and constricted cells.

**Conclusions:**

Taken together, these results suggest that gradients are a robust and potentially common mechanism for providing intracellular spatial cues.

## Background

Morphogen gradients form the basis of development in eukaryotes and particularly in embryos, where the spatial distribution of molecules such as maternal mRNAs can give rise to organism-wide properties, transferring information across several orders of magnitude in space [1]. Asymmetries have also been identified in bacterial cells, which exhibit sophisticated and highly dynamic spatial organization essential to many key processes such as chemotaxis, chromosome organization, DNA replication, and cell division [2,3]. In the rod-shaped bacterium *Pseudomonas aeruginosa*, the second messenger cyclic-di-GMP is asymmetrically distributed between the daughter cells, being about four times as abundant in the non-flagellated compared with the flagellated cell, where it may regulate pili biosynthesis to promote surface adhesion [4]. In *Shigella flexneri,* polar localization of the virulence protein IcsA is maintained through polar export and then uniform cleavage by the outer membrane protease IcsP, a scenario reminiscent of the localized synthesis and uniform decay that can generate morphogen gradients in eukaryotic embryos [5-7]. In *Escherichia coli*, chemotaxis is controlled by localized signaling proteins [8,9]. Disruption of the localization of one of the components in this system gives rise to a gradient of the phosphorylated form of the response regulator CheY; this gradient leads to spatial cues that cause motors in different parts of the cell to rotate in different directions [9].

One paradigm for the establishment of intracellular asymmetries in bacteria that has emerged is asymmetric localization of components in a signaling pathway. The bacterium *Caulobacter crescentus* is a model system for studying asymmetric localization, with 10% of its genes encoding proteins that are non-uniformly localized [10]. Each cell division in *C. crescentus* is asymmetric, leading to two cell types: a motile swarmer cell in S phase and a sessile stalked cell in G1 phase. Cell fate in *C. crescentus* is controlled by the cytoplasmic master regulator CtrA, an essential transcription factor that, in its phosphorylated form, binds to and silences the origin of replication. In previous work, we demonstrated that the bifunctional, polarly localized kinase CckA gives rise to a gradient of the phosphorylated form of CtrA by acting as a CtrA-phosphate source and sink at the two poles and thereby establishing replicative asymmetry in the pre-divisional cell [11]. The CtrA-CckA system is one of many examples of localization in a two-component system, in which a histidine kinase (CckA in this case) is regulated by external stimuli, and generates a response by phosphorylating or dephosphorylating a cognate response regulator (CtrA) [12-14].

The establishment of intracellular phosphorylation gradients has been previously explored computationally and theoretically in the context of eukaryotic cells, with previous results illustrating that a membrane-bound kinase and a cytoplasmic phosphatase can give rise to a steady-state cytoplasmic gradient in a spherical cell, similar to the origins of many morphogen gradients [13,14]. For a scenario in which the kinase and phosphatase are bound to two separate locations in the cell, gradients in the levels of the two phosphorylation states of the protein can be achieved while maintaining a uniform total protein level [13,14]. When the diffusion constant differs between the phosphorylated and unphosphorylated species, for instance due to the binding of another protein in the phosphorylated state, a gradient in the total amount of protein can also occur [15].

A great deal of attention has also been devoted to how bacteria sense extracellular gradients through processes such as chemotaxis [16]. However, a comprehensive study of the formation and maintenance of *intracellular* spatial gradients in bacteria has not yet been undertaken, despite the physiological relevance of gradients in bacterial development. It has often been assumed that the short time scales associated with diffusion inside a micron-sized bacterium preclude the establishment of significant spatial gradients of cytoplasmic proteins. Given the typical size of a bacterium and cytoplasmic diffusion rates, is localized regulation of phosphorylation state an effective mechanism for the production of a gradient?

Here, we use mathematical modeling of reaction-diffusion systems to probe the biochemical requirements for gradient formation. We also investigate how gradients are affected by physical constraints imposed by other cellular processes such as growth and division. Our results encompass a general description of the spatiotemporal dynamics of a substrate subject to regulation by localized sources and sinks, proteolysis, fluctuating concentrations and growth. We find that localized sources and sinks can produce gradients that are robust to most perturbations. We also consider a number of different cell morphologies and localization phenotypes to mimic biologically relevant morphological changes. Our analysis demonstrates that most typical cell shapes and sizes can support gradient formation, suggesting that this may be a common mechanism for providing intracellular spatial cues.

## Results

### Physical constraints on the establishment of spatial gradients in bacteria

To elucidate the general kinetic and physical constraints on gradient formation, we first consider a simple model of a rod-shaped cell with a source and a sink localized at opposite ends. We use phosphorylation in bacterial two-component systems as a case study; the same analysis can be applied to a source and sink of any other chemical modification with little or no change. In a two-component system, there is a source or sink for the phosphorylated form of the response regulator, R ∼ P [12-14]. We consider a scenario in which a kinase (source) and phosphatase (sink) are localized at opposite poles and we analyze the diffusion of the response regulator along the long axis of a rod-shaped cell extending from *x* = 0 to *x* = *L*. We denote the spatial- and time-dependent densities of the two phosphorylation states of the response regulator as [R] (*x*,*t*) and [R ∼ P] (*x*,*t*), and assume that the diffusion constant *D* is independent of phosphorylation state. We assume that R is phosphorylated at the left boundary of the cell (*x* = 0) at a rate *σ*_*k*_, and R ∼ P is dephosphorylated at the right boundary (*x* = *L*) at a rate *σ*_*p*_. We first ignore synthesis and degradation, such that the total number of molecules is fixed at R_tot_. The reaction-diffusion equations describing the dynamics of [R] and [R∼P] are then

(1) $$\begin{array}{ccc} \;\frac{\partial [R]}{\mathrm{\partial t}} & = & D\frac{\partial^{2}[R]}{\partial x^{2}}-l(x)\sigma_{k}[R]+r(x)\sigma_{p}[\text{R}∼\text{P}] \end{array}$$

(2) $$\begin{array}{ccc} \;\frac{\partial [R∼P]}{\mathrm{\partial t}} & = & D\frac{\partial^{2}[\text{R}∼\text{P}]}{\partial x^{2}}+l(x)\sigma_{k}[R]-r(x)\sigma_{p}[\text{R}\;∼\;\text{P}], \end{array}$$

where *l*(*x*) = *δ*(*x*) and *r*(*x*) = *δ*(*x* − *L*) are delta functions that define the left and right poles, respectively. In later analyses, we will also employ functions *l*(*x*) and *r*(*x*) that are nonzero across a spatially extended region representing poles of size *r*_*p*_.

Between the two poles, where *l*(*x*) = *r*(*x*) = 0, Eqs. 1 and 2 both reduce to the diffusion equation without reactions and hence the steady-state solutions are linear and can be written as [R](*x*) = *Ax* + *B*and [RcP] (*x*) =*A*_*Px*_ + *B*_*P*_. Because there are reactions occurring at the two poles, acting as sources and sinks for R and R ∼ P, the slopes *A* and *A*_*P*_need not be zero. The no-flux (mass-conserving) boundary conditions at the two poles impose the constraints

$$\begin{array}{c} D{\left(\frac{\partial [R]}{\mathrm{\partial x}}\right|}_{x=0}=\sigma_{k}[R](0)\\ D{\left(\frac{\partial [R]}{\mathrm{\partial x}}\right|}_{x=L}=\sigma_{p}[\text{R}\;∼\;\text{P}](L)\\ D{\left(\frac{\partial [\text{R}\;∼\;\text{P}]}{\mathrm{\partial x}}\right|}_{x=0}=-\sigma_{k}[R](0)\\ D{\left(\frac{\partial [\text{R}\;∼\;\text{P}]}{\mathrm{\partial x}}\right|}_{x=L}=-\sigma_{p}[\text{R}\;∼\;\text{P}](L), \end{array}$$

which are equivalent to *DA* = *σ*_*k*_*B*_*P*_ = *σ*_*p*_(*A*_*P*_*L* + *B*_*P*_) = − *DA*_*P*_, indicating that [R] and [R∼P] have opposite slopes and that their sum is a constant *B* + *B*_*P*_independent of *x*. This constant sum indicates that the overall protein distribution, as would be experimentally measured by tagging the response regulator with a fluorescent protein, would be uniform. This was indeed the case in the CtrA-CckA system, in which a uniform distribution of CtrA-YFP was observed experimentally despite the existence of gradients of the active, phosphorylated form CtrA∼P [11]. However, if the diffusion constant is not the same in the two phosphorylation states, then *A* will be different from −*A*_*P*_ by the ratio *D*_*P*_/*D* and the overall protein distribution will not be uniform.

Using the constraint on the total number of molecules

$$R_{\text{tot}}=\int_{0}^{L}\left(\left[R]+[\text{R}\;∼\;\text{P}\right]\right)\mathrm{dx}=\frac{L^{2}}{2}(A+A_{P})+L(B+B_{P}),$$

the steady-state solutions can be written in terms of the diffusion time scale *τ*_*D*_=*L*^2^ / *D* and the source (sink) time scales *τ*_*k*_ = 1/*σ*_*k*_(*τ*_*p*_ = 1/*σ*_*p*_) as

(3) $$\begin{array}{ccc} A & = & \frac{R_{\text{tot}}}{D}\frac{1}{\tau_{k}+\tau_{p}+\tau_{D}}=-A_{P} \end{array}$$

(4) $$\begin{array}{ccc} B & = & \frac{R_{\text{tot}}}{L}\left(1-\frac{\tau_{p}+\tau_{D}}{\tau_{k}+\tau_{p}+\tau_{D}}\right) \end{array}$$

(5) $$\begin{array}{ccc} B_{P} & = & \frac{R_{\text{tot}}}{L}\frac{\tau_{p}+\tau_{D}}{\tau_{k}+\tau_{p}+\tau_{D}}. \end{array}$$

These equations immediately indicate the strength of the gradient produced for a given *σ*_*k*_ and *σ*_*p*_: for a slow source and sink such that *τ*_*k*_ + *τ*_*p*_≫*τ*_*D*_, the normalized slope *D*_*τD*_*A*/R_tot_ is much less than 1, indicating a weak gradient. In contrast, as *τ*_*k*_ + *τ*_*p*_ crosses below the diffusive time scale *τ*_*D*_, the slope approaches a maximum value of *A* = R_tot_/*L*^2^ with [R](0) =[R ∼ P](*L*) = 0 and [R](*L*) =[R∼P](0) = R_tot_/*L*. Thus, both *τ*_*k*_ and *τ*_*p*_ must be less than *τ*_*D*_to produce a substantial gradient. For a 70 kDa protein diffusing in water, the Einstein relation (*D* = *k*_*B*_*T* /6*π μR*) provides an estimate of *D*∼2*μm*^2^ /s, similar to the measured diffusion constant of a maltose-binding protein in *E. coli*[17,18]. For the simulations that follow, we assume *D* = 2*μm*^2^ /s and note that changing *D* will simply adjust the time scale *τ*_*D*_ to which all kinetic rates should be compared. For *D* = 2 *μm*^2^ /s, the time scale for diffusion between the poles of a 2 *μ*m-long bacterium is *τ*_*D*_ = 1 s, and so we require the source and sink rates to be faster than 1/*τ*_*D*_∼1/s in order that phosphorylation can outcompete the uniformity produced by diffusion.

To quantitatively compare the density produced by an asymmetrically localized source and sink to other potential gradient-generation mechanisms, we focus on the phosphorylated (assumed to be active) form and define a metric *η* that is the ratio of R∼P between two regions extending a distance *Δ*from the left and right poles (as we will show, the slope need not be constant for other scenarios involving, e.g. synthesis and degradation, and hence the slope is not always a well-defined scalar metric). The ratio *η*calculated using Eqs. 3 and 5 is

(6) $$\eta =\frac{L-\Delta /2+D/\sigma_{p}}{\Delta /2+D/\sigma_{p}},$$

which has a maximum value of 15 for *Δ* = *L*/8, the approximate value for a rod-shaped bacterial cell such as *E. coli* or *C. crescentus,* and _*σp*_→*∞*. Note that *η* is independent of *σ*_*k*_, which determines the total levels of R∼P but scales both poles equally and hence does not affect the ratio.

**Figure 1:**
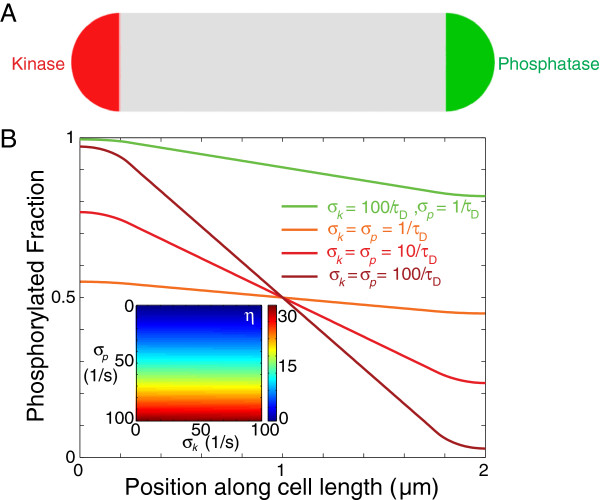
**A phosphorylation gradient can be produced by fast asymmetric source and sink activities.A**) Schematic of localization of source and sink. **B**) Mathematical modeling of the spatial asymmetry in phosphorylated response regulator for different source and sink rates. A substantial gradient is obtained only when the phosphorylation rate _*σk*_and dephosphorylation rate _*σp*_are faster than the inverse of the time scale required for diffusion across the cell, 1/*τ*_*D*_ = 2*D*/*L*^2^. We model the cell in 1D with length *L* = 2 *μ*m and poles of extent *r*_*p*_ = 0.25 *μ*m. The diffusion constant is *D* = 2 *μ*m^2^/*s*. (Inset) The ratio of R∼P between the two poles *η*is virtually independent of the source rate *σ.*_*k*_

In the analyses that follow, we wish to account for the dimensions of the cell poles and hence we modify the polar functions to be *l*(*x*) = 1 for *x* < *r*_*p*_ and 0 elsewhere, and *r*(*x*) = 1 for *x* > *L* − *r*_*p*_and 0 elsewhere. In Figure 1, we numerically solve Eqs. 1 and 2 for various values of *σ*_*k*_ and *σ*_*p*_ in a cell of length *L* = 2 *μ*m and radius *r*_*p*_ = 0.25 *μ*m, mimicking the dimensions of a *C. crescentus* cell. The steady-state distribution of R ∼ P has the expected linear behavior between the two poles (Figure 1A), with a significant gradient only when *σ*_*k*_*σ*_*p*_≫ 1/*τ*_*D*_. The densities flatten out at the poles due to the source and sink activities not being point sources, nevertheless the polar [R ∼ P] ratio *η* remains virtually independent of *σ*_*k*_(Figure 1, inset). Phosphotransfer rates in biological systems span several orders of magnitude and have been reported to be as high as 800/s, as in the phosphorylation of CheY by the polarly clustered kinase CheA [19]. Given such fast kinetics, spatial gradients are therefore a biologically relevant mechanism by which bacterial cells can produce intracellular asymmetries.

### Effects of localized synthesis and degradation on gradient formation

Given that changes in activity of the source or sink can enhance or reduce the gradient, we next explored the extent to which synthesis and degradation can affect a gradient. Since dephosphorylation and degradation have similar effects on the R ∼ P density, we sought to address whether localized proteolysis could enhance the gradient by selectively removing protein. We assume that proteolysis of R and R ∼ P occurs at a rate *γ*(*x*) that may be spatially dependent but is independent of phosphorylation state. In order that proteolysis not completely deplete the entire pool of proteins, we assume that it is balanced by synthesis of unphosphorylated substrate at a rate *α*(*x*):

(7) $$\begin{array}{ll} \frac{\partial [R]}{\mathrm{\partial t}}=D\frac{\partial^{2}[R]}{\partial x^{2}} & -l(x)\sigma_{k}[R]+r(x)\sigma_{p}[\text{R}\;∼\;\text{P}]\\ & -\gamma (x)[R]+\alpha (x) \end{array}$$

$$\begin{array}{ll} \frac{\partial [\text{R}\;∼\;\text{P}]}{\mathrm{\partial t}}=D\frac{\partial^{2}[\text{R}\;∼\;\text{P}]}{\partial x^{2}} & \;+l(x)\sigma_{k}[R]-r(x)\sigma_{p}[\text{R}\;∼\;\text{P}]\\ & -\gamma (x)[\text{R}\;∼\;\text{P}]. \end{array}$$

If synthesis is uniform (*α*independent of *x*), we can rescale Eqs. 7 and 8 to obtain equations for the normalized concentrations $[\tilde{R}]=[R]/\alpha$ and $\tilde{\left[\text{R}\;∼\;\text{P}\right]}=[\text{R}\;∼\;\text{P}]/\alpha$ that are independent of *α*:

$$\begin{array}{ll} \frac{\partial \tilde{\left[R\right]}}{\mathrm{\partial t}}=D\frac{\partial^{2}\tilde{\left[R\right]}}{\partial x^{2}} & -l(x)\sigma_{k}\tilde{\left[R\right]}+r(x)\sigma_{p}\tilde{\left[\text{R}\;∼\;\text{P}\right]}\\ & -\gamma (x)\tilde{\left[R\right]}+1 \end{array}$$

$$\begin{array}{ll} \frac{\partial \tilde{\left[\text{R}\;∼\;\text{P}\right]}}{\mathrm{\partial t}}=D\frac{\partial^{2}\tilde{\left[\text{R}\;∼\;\text{P}\right]}}{\partial x^{2}} & \;+l(x)\sigma_{k}\tilde{\left[R\right]}-r(x)\sigma_{p}\tilde{\left[\text{R}\;∼\;\text{P}\right]}\\ & -\gamma (x)\tilde{\left[\text{R}\;∼\;\text{P}\right]}. \end{array}$$

The steady-state solutions for $\tilde{\left[R\right]}$ and $\tilde{\left[\text{R}\;∼\;\text{P}\right]}$ represent functions such that the ratio *η* evaluated using $\tilde{\left[\text{R}\;∼\;\text{P}\right]}$ will be identical to the ratio evaluated using [R ∼ P] for any synthesis rate *α*. This equivalence results because each response regulator molecule independently interacts with the source and sink, hence the steady-state densities reflect the probability of each molecule being found at a particular location in a given phosphorylation state and thus the ratio *η*is independent of the overall protein levels, which are dictated by the synthesis rate *α*. We note that we have ignored any potential saturation of the source and sink that would occur at high R_tot_; we will address this saturation below.

**Figure 2:**
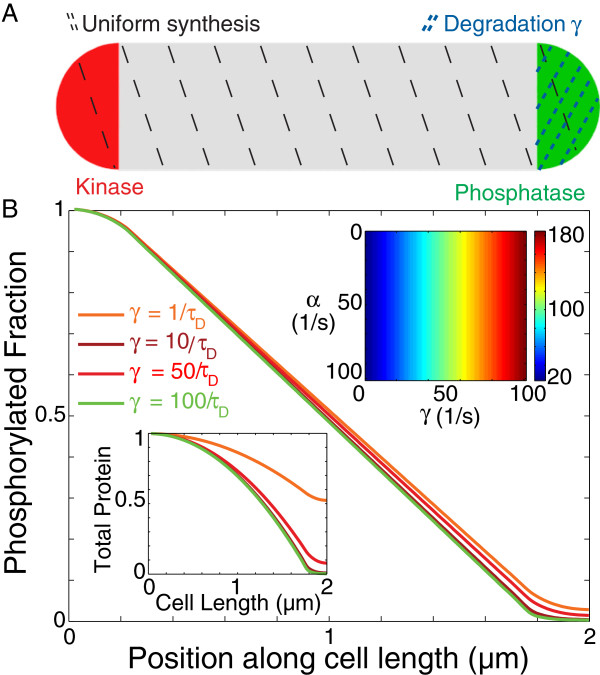
**Localized degradation does not significantly affect to the R ∼ P gradient even at high proteolysis rates.A**) Schematic of the localization of source and sink. Synthesis occurs throughout the cell, whereas degradation occurs only at the phosphatase pole. **B**) Mathematical modeling of the effects of proteolysis on the distributions of R ∼ P for *α* = 1/*τ*_*D*_. (Left inset) The total amount of substrate is not uniform. (Right inset) The ratio of R ∼ P between the two poles is independent of the synthesis rate.

Although polarly localized proteolysis could selectively deplete [R ∼ P] at the pole with the higher concentration, we have previously shown that the same constraints determined for phosphorylation kinetics in Sec. 1.1 also apply to localized proteolysis [11]. That is, significant gradients arise only when the proteolysis rate *γ* is fast relative to 1/*τ*_*D*_; in that case, the total response regulator protein levels would show a gradient in addition to the gradient of R ∼ P (Figure 2, left inset). However, in *E. coli,* less than 10% of proteins were found to be degraded in less than one hour [20]. Moreover, the average protein synthesis rate is 10-20 amino acids/s [21], indicating that a protein of typical size ∼300 amino acids would require on the order of minutes for synthesis to complete. Therefore it is likely that both synthesis and degradation rates are much slower than diffusion [17]. Under these conditions, synthesis and degradation have little effect on the gradient even if mRNAs or the proteolytic complex are localized, since diffusion will distribute the response regulator proteins in the cell faster than synthesis or degradation occurs. Therefore diffusion in micron-sized organisms dominates over the slower processes of synthesis and degradation, making these processes unlikely to generate or disrupt dynamic intracellular gradients or asymmetries.

### Effects of enzyme saturation on gradient formation

If the levels of the substrate are considerably higher than the levels of the source and sink, the saturation of their activities could effectively decrease the rates of phosphorylation and dephosphorylation and thereby affect gradient establishment. To address the amount of saturation required to perturb a gradient, we modified the rates of phosphorylation and dephosphorylation to reflect Michaelis-Menten kinetics through the effective rates *σ*_*k*,*e*_,*σ*_*p*,*e*_:

(8) $$\sigma_{p,e}[R∼P]=\sigma_{p}[P_{0}]\frac{\left[\text{R}\;∼\;\text{P}\right]}{K_{m}+[\text{R}\;∼\;\text{P}]}$$

(9) $$\sigma_{k,e}[R]=\sigma_{k}[K_{0}]\frac{\left[R\right]}{K_{m}+\left[R\right]},$$

where *σ*_*k*_(*σ*_*p*_) is the unsaturated enzymatic rate, [K_0_][P_0_] is the polar kinase (phosphatase) concentration and *K*_*m*_is the substrate concentration at which the reaction rate is half of *σ*_*k*_[K_0_](*σ*_*p*_[P_0_]). We choose *K*_*m*_/[R_tot_] = 2 to consider a regime of high sensitivity to the substrate concentration at the pole.

**Figure 3:**
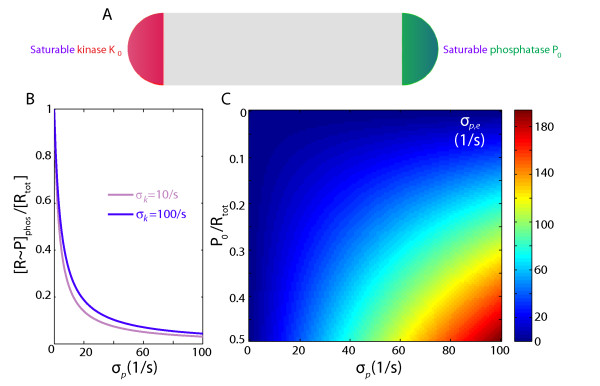
**Source and sink saturation does not significantly reduce the R ∼ P gradient except in very stringent saturation conditions.A**) Schematic of localization of source and sink. **B**) Mathematical modeling of the R ∼ P concentration near the phosphatase pole [R ∼ P]_phos_normalized to the total response regulator concentration [R_tot_] for varying *σ*_*p*_, and *σ*_*k*_ = 10 and 100/s. **C**) The effective phosphatase rate *σ*_*p*,*e*_is high enough to outcompete diffusion except when the number of enzymes is less than 10% than of the substrate.

Unlike *in vitro* experiments, where the substrate is assumed to be uniform in space, in our system [R ∼ P] varies as a function of position along cell length, as seen in a previous section. We are interested in the R ∼ P concentration only where it may interact with the saturable enzyme, namely at the phosphatase pole (_[R ∼ P]phos_); the following arguments are similarly applicable to considerations of [R] at the kinase pole. [R ∼ P]_phos_ levels vary strongly with *σ*_*p*_ and less so with *σ*_*k*_ (Figure 3B) so that we can approximate Eq. 9 as

$$\sigma_{p,e}(\sigma_{p},[P_{0}])≃\frac{\sigma_{p}[P_{0}]}{K_{m}+{\left[\text{R}\;∼\;\text{P}(\sigma_{p})\right]}_{\mathrm{phos}}}.$$

Figure 3C shows the effective rate for a cell with poles of extent *r*_*p*_ = *L*/8, for varying levels of phosphatase _P0_ (normalized by the total number of response regulator proteins R_tot_) and *σ*_*p*_. At high P_0_ levels compared with R_tot_, the levels of R ∼ P_p*hos*_ are too low to saturate the enzyme, and *σ*_*p*,*e*_≃*σ*_*p*_[P_0_]. As long as the amount of enzyme is in excess of 10% of that of the substrate (P_0_/R_tot_ > 0.1), a gradient can be established, since the effective phosphatase rate exceeds the diffusion rate.

### Effect on delocalized activities on gradient formation

Thus far we have assumed that the substrate is phosphorylated and dephosphorylated only at the poles by its specific partner source and sink, respectively. However, *in vitro* phosphorylation measurements have shown that crosstalk with other sources can occur on long time scales [22]. To determine the extent to which crosstalk would affect the establishment of a gradient, we added spatially uniform phosphorylation and dephosphorylation activities to Eqs.1 and 2 with rates $\sigma_{k}^{0}$ and $\sigma_{p}^{0}$, respectively:

(10) $$\begin{array}{ll} \frac{\partial [R]}{\mathrm{\partial t}}=D\frac{\partial^{2}[R]}{\partial x^{2}} & -l(x)\sigma_{k}[R]+r(x)\sigma_{p}[\text{R}\;∼\;\text{P}]-\sigma_{k}^{0}[R]\\ & +\sigma_{p}^{0}[\text{R}\;∼\;\text{P}] \end{array}$$

(11) $$\begin{array}{ll} \frac{\partial [\text{R}\;∼\;\text{P}]}{\mathrm{\partial t}}=D\frac{\partial^{2}[\text{R}\;∼\;\text{P}]}{\partial x^{2}} & \;+l(x)\sigma_{k}[R]-r(x)\sigma_{p}[\text{R}\;∼\;\text{P}]\\ & +\sigma_{k}^{0}[R]-\sigma_{p}^{0}[\text{R}\;∼\;\text{P}]. \end{array}$$

In the absence of polar activity (*σ*_*k*_=*σ*_*p*_=0), the nonspecific activities define the steady-state levels [R]_0_and [R ∼ P]_0_ such that ${\left[R\right]}_{0}/{\left[\text{R}\;∼\;\text{P}\right]}_{0}=\sigma_{p}^{0}/\sigma_{k}^{0}$.

**Figure 4:**
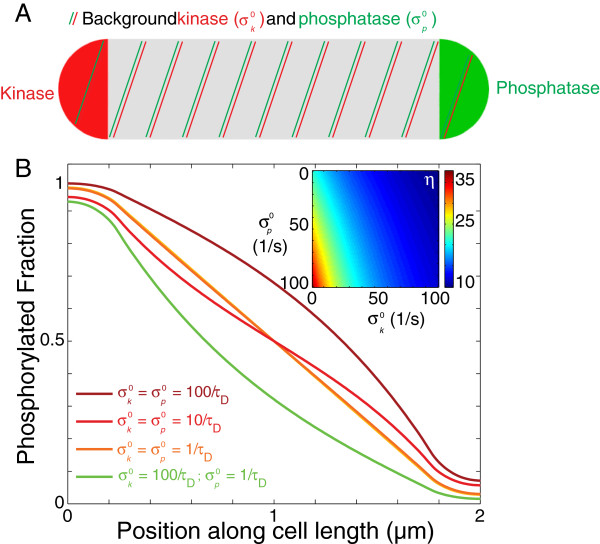
**Background source and sink activities do not significantly perturb the R ∼ P gradient even for high rates.A**) Schematic of localization of source and sink activities; the green and red shading represent the background activity. **B**) Mathematical modeling of the effects of background source and sink activities on the distributions of [R ∼ P]. (Inset) The ratio of [R ∼ P] between the two poles is not significantly affected by background source and sink rates.

The addition of crosstalk to the polarly localized activities in Figure 1 with *σ*_*k*_ = *σ*_*p*_=100/s modified the shape of the steady-state concentration profiles, with a more concave or convex shape if the source or sink crosstalk was dominant, respectively (Figure 4). However, even at very high rates $\sigma_{k}^{0}$ and $\sigma_{p}^{0}$ comparable to *σ*_*k*_ and *σ*_*p*_, the localized sink and source ensured that a gradient is maintained. For extremely rapid uniform source activity, the [R ∼ P] profile was somewhat flattened, but the polar ratio *η* was reduced by only by a small factor (<4, Figure 4, inset). Moreover, uniform sink activity actually enhances the polar ratio due to the greater fractional reduction in [R ∼ P] near the sink pole (Figure 4, inset). Given the slow crosstalk time scales on the order of tens of minutes to an hour observed *in vitro*[22], it is likely that nonspecific activities has a negligible effect on the ability of localized source and sink activity to establish a gradient.

### Effect of cell length on gradient formation

As a cell elongates, the boundary conditions on the reaction-diffusion equations in Eqs. 1,2 change. Given that the establishment of a gradient is dictated primarily by the relative comparison of the diffusive time scale between the two poles (*τ*_*D*_∼*L*^2^/2D) and the source/sink rates, we expect gradient establishment to be facilitated by the quadratic increase in *τ*_*D*_ as *L* increases. That is, as the cell doubles in length, the ratio of *σ*_*k*_or *σ*_*p*_ to 1/*τ*_*D*_will increase by a factor of 4, expanding the regime of rates that satisfy *σ*_*k*_,*σ*_*p*_ ≫ 1/*τ*_*D*_.

**Figure 5:**
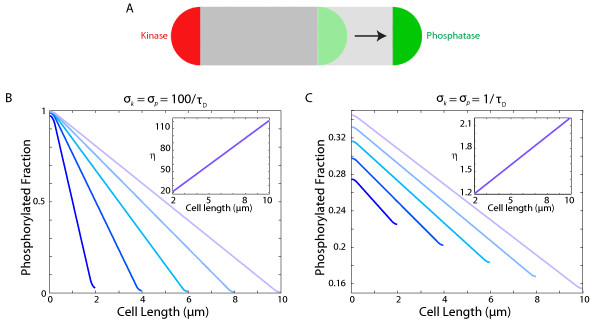
**Elongation enhances spatial asymmetryA**) Schematic of localization of source and sink. **B**) Mathematical modeling of the effects of varying cell length on the distribution of [R ∼ P] for *σ*_*k*_ = *σ*_*p*_ = 100/*τ*_*d*_. (Inset) The ratio of [R ∼ P] between the two poles increases linearly with increasing cell length. **C**) At enzymatic rates close to the diffusion rate (*σ*_*k*_=*σ*_*p*_=1/*τ*_*d*_), the difference in [R ∼ P] between the poles increases with cell length; the polar ratio also increases linearly with cell length however it is not affected as significantly as the difference between the poles (inset).

For sufficiently fast *σ*_*k*_,*σ*_*p*_(100/s), the polar ratio *η*is enhanced and scales approximately linearly with *L* (see Eq. 6) (Figure 5A, inset), even though the slope decreases as the cells elongates (∝1/*L*). This scaling of *η*is due to the decrease in the ratio between the size of the poles relative to the cell length, which creates a more pronounced depletion close to the sink and enhances the density near the source; the increase in *η* is primarily due to depletion at the swarmer pole. Nonetheless, the polar values are roughly the same at any cell length, due to the fast enzymatic rates. In contrast, for slow *σ*_*k*_,*σ*_*p*_(1/s), cell growth strongly increases the absolute difference in R ∼ P concentration between the poles. As seen in Figure 5B, the R ∼ P difference between the two poles increases dramatically from about 20% at L=2 *μ*m, to over a two-fold change at L=10 *μ*m.

Thus, in physiological conditions, once a gradient is established, cell elongation enhances asymmetry in R ∼ P. Moreover, for small *σ*_*k*_or *σ*_*p*_, there might not be a significant gradient until the cell grows past a length *L* such that the diffusion rate 1/*τ*_*D*_ can be overcome by the source and the sink.

### Effect of cell geometry on gradient formation

Thus far we have focused on one-dimensional diffusion to illustrate the factors that influence gradients in rod-shaped cells. However, the three-dimensional geometry of cells could potentially impact the consequences of an asymmetric source and sink localization pattern. In addition to rod-shaped species such as *E. coli*, bacteria can adopt a wide variety of morphologies, including crescents, spheres, disks, and branched cells [23]. To investigate how morphology affects gradient formation, we studied our reaction-diffusion model in three-dimensional (3D) geometries (see Methods) mimicking several typical shapes and shape transitions. In 3D, the diffusion time scale is determined by the longest relevant length scale separating the source and the sink. In the simplest case in which source and sink are oppositely localized at the poles of a rod of length *L*, the relevant time scale is approximately $\tau_{D}∼L^{2}/2D$.

To keep our analysis consistent with the 1D modeling discussed above we maintained the diffusion constant *D* = 2 *μ*m^2^/s and distributed the kinase and phosphatase activities on the cell membrane at opposite poles, maintaing a constant active surface area across all 3D simulations. We also appropriately scaled the relevant enzymatic activities *σ*_*k*_ and *σ*_*p*_, such that the total number of active kinases and phosphatases remained consistent. This adaptation of the kinase and phosphatase rates to 3D allows for a direct comparison of all geometries and across dimensions.

#### Gradient formation in a crescent-shaped cell

**Figure 6:**
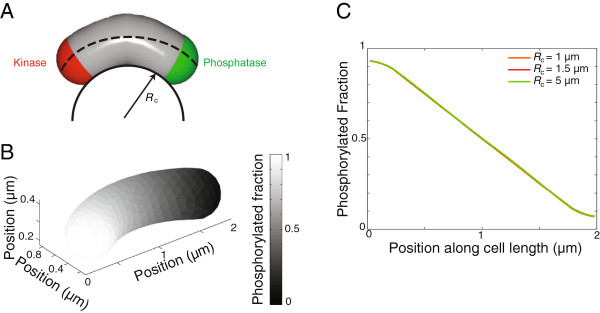
**Cell bending does not significantly affect the R ∼ P gradient even for small cellular radius of curvature.A**) Schematic of a bent cell with radius of curvature *R*_*c*_with oppositely localized kinase and phosphatase. **B**) 3D mathematical modeling of the distribution of [R ∼ P] in a bent cell with radius of curvature *R*_*c*_ = 1 *μ*m. **C**) [R ∼ P] line scan through the cell middle showing volume-weighted average density along cell length (along the dashed line in A) for different *R*_*c.*_

Several model organisms such as *C. crescentus* and *Vibrio cholerae* have the shape of a curved rod (or crescent) [23]. Moreover, rod-shaped *E. coli* cells confined in curved micro-chambers grow into curved rods with a curvature matching that of the chamber [24]. To investigate effects of the crescent-shaped geometry, we modeled a *C. crescentus* cell as curved cylinder with a radius of curvature *R*_*c*_and assumed that the sink and source were oppositely localized at the hemispherical poles (Figure 6A). Even when the cellular radius of curvature was similar to the cell width (*R*_*c*_ = 1 *μ*m), we found that the curved geometry did not have a noticeable impact on gradient formation. Thus, the curvature of a crescent-shaped cell can be ignored when considering gradient formation mechanisms and gradient formation in a rod-shaped cell can be considered accurately in 1D.

#### Gradient formation in round cells

**Figure 7:**
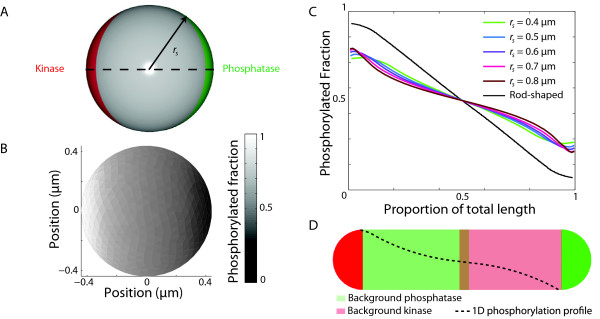
**Spherical cells can support gradients along the source/sink axis.A**) Schematic of a spherical cell of radius *r*_*s*_with oppositely localized kinase and phosphatase spread over a spherical cap. **B**) 3D mathematical modeling of the distribution of [R ∼ P] in a spherical cell with radius *r*_*s*_ = 0.4 *μ*m. **C**) [R ∼ P] line scan through the cell middle showing average volume-weighted density along the cell length (along the dashed line in **A**) for different radii *r*_*s*_. **D**) Schematic of 1D distributions of kinase and phosphatase activities that produce profiles mimicking those in (**C**) for *r*_*s*_> 0.5 *μ*m, with background kinase and phosphatase activities localized from the middle of the cell to the phosphatase pole and kinase pole, respectively, and overlapping by *L*/20.

Many important model bacteria, such as *Streptococcus pneumoniae, Staphylococcus aereus,* and the cyanobacterium *Synechocystis* sp. PCC6803, are cocci (round). Furthermore, other typically non-round species such as *E. coli*, *Arthrobacter globiformis, Acinetobacter baumannii*, and *Rhodococcus equi* become round in specific conditions or in specific growth phases such as stationary phase [23]. To investigate whether gradients can arise in round cells, we modeled the effects of sinks and sources oppositely localized to the poles of spheres of different radii. Our simulations predicted that spherical cells can also support gradients (Figure 7B), although their spatial dependence was dependent on the cell radius (Figure 7C). In particular, the average density weighted by the volume of each finite element of R ∼ P along the line connecting the two poles flattened with increasing radius, while close to the source and sink the slope increased (Figure 7C). The reduced slope away from the poles is due to the increased volume into which the response regulator molecules can diffuse, decreasing the probability of binding to the kinase or the phosphatase poles and therefore change phosphorylation state.

Using the understanding generated by our 1D simulations, we noted that the flattening of the density near the equator was similar to the effect of background kinase and phosphatase activities in Figure 4. Indeed, we were able to recapitulate density profiles mimicking the curves in Figure 7C in 1D simulations by introducing kinase and phosphatase activities ( $\sigma_{k}^{0},\sigma_{p}^{0}∼1-10/s$) localized from the middle of the cell to the phosphatase pole and kinase pole, respectively, and overlapping by a small amount near midcell (*L*/20) (Figure 7D). In particular phosphatase (kinase) activity at the kinase (phosphatase) pole caused the observed non-linear shape of the density profile.

#### Gradient formation in disk-shaped cells

**Figure 8:**
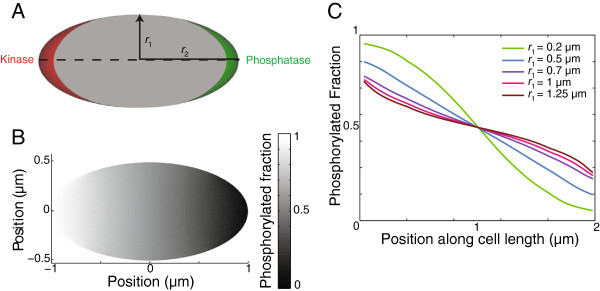
**Disk-shaped cells can support gradients along the source/sink axis.A**) Schematic of a disk-shaped cell with distance *r*_2_ = 1 *μ*m from the center to the poles and equatorial radius *r*_1_ between 0.2 and 1.25 *μ*m, with oppositely localized kinase and phosphatase. **B**) 2D Mathematical modeling of the distribution of [R ∼ P] in an ellipse geometry. **C**) [R ∼ P] line scan through the cell middle showing volume-weighted average density along cell length (along the dashed line in A) for varying ellipticity.

Rod-shaped bacteria such as *E. coli* and *B. subtilis* can penetrate channels whose width is as small as half the diameter of the cells, producing thin and wide disk-shaped cells [25]. To investigate gradient formation in disk-shaped cells, we studied our reaction-diffusion model in a 2D ellipse geometry in which we ignored diffusion along the shortest (perpendicular) dimension, with oppositely localized source and sink. We varied the equatorial radius *r*_1_ between 0.2 and 1.25 *μ*m while keeping the distance from the center to the poles *r*_2_ = 1*μ*m (Figure 8A). The gradients of [R ∼ P] in disk-shaped cells were similar to those of spherical cells (Figure 7) for *r*_1_ >=*r*_2_ and to the rod-shaped cells (Figure 6) for *r*_1_ < *r*_2_ (Figure 8C). These results show that cells can preserve asymmetries and gradients even when deformed to have a high aspect ratio.

#### Gradient formation in branched cells

**Figure 9:**
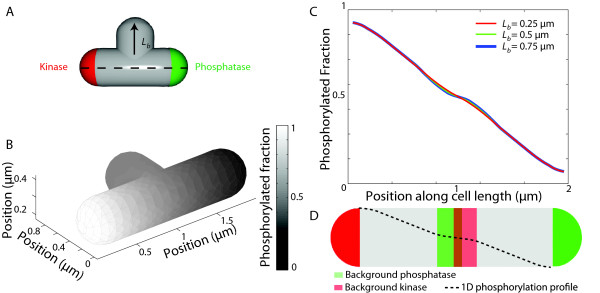
**Spatial asymmetry in [R ∼ P] is not sensitive to branching.A**) Schematic of a branched cell with oppositely localized kinase and phosphatase in the rod-shaped region of the cell. The branch occurs at midcell at a right angle to the rest of the cell, and has length *L*_*b*_. **B**) 3D mathematical modeling of the distribution of [R ∼ P] in a branched cell with *L*_*b*_ = 0.5 *μ*m. **C**) [R ∼ P] line scan through the cell middle showing volume-weighted average density along cell length (along the dashed line in A) for varying branch lengths. **D**) Schematic of 1D distribution of kinase and phosphatase activities that produce profiles mimicking those in (**C**) for *L*_*b*_ > 0.5 *μ*m, with background kinase and phosphatase localized at the middle of the cell and overlapping by *L*/10.

Several bacterial species grow as branched rods in the absence of particular nutrients or in response of environmental cues. In particular the bifid, or Y-shaped morphology, is common in species such as *Lactobacillus bifidus,* where wild type cells grow as branches, as well as in mutants of rod-shaped species such as *E. coli*[23]. To investigate the effect of a branch on a gradient, we considered a rod-shaped cell with oppositely localized sink and source and a protrusion at mid-cell of various lengths (*L*_*b*_). Similar to simulations involving a spherical cell with large radius (Figure 7C), a branch slightly decreased the steepness of the gradient near the branching location, but otherwise had little effect on the linear gradient produced in the rod-shaped cell compartment (Figure 9B,C). Furthermore, the distribution of [R ∼ P] was fairly uniform along the branch.

Similar to simulations in spherical cells (Figure 7D), we could recapitulate density profiles in the rod-shaped compartments in 1D simulations from Figure 9C by adding non-uniform kinase and phosphatase activities ( $\sigma_{k}^{0},\sigma_{p}^{0}∼10/s$) centered in a short interval near midcell, with the kinase activity shifted slightly toward phosphatase pole and phosphatase activity slightly toward kinase pole (Figure 9D).

Therefore, even the growth of a branch comparable in length to the rest of the cell length does not significantly affect gradient formation.

#### Gradient maintenance during cell division

During cell division, the septum provides a barrier to diffusion. For Gram-negative rod-shaped organisms such as *E. coli* or *C. crescentus*, the division machinery progressively constricts the midcell region to form partial hemispheres that eventually become the new poles of the daughter cells. As the amount of constriction increases, response regulator molecules are less likely to diffuse from one side of the cell to the other and hence are less likely to switch phosphorylation state. When cytokinesis completes, the source and the sink are completely separated and can no longer produce a gradient.

**Figure 10:**
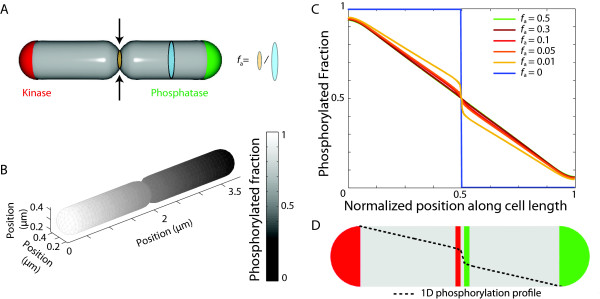
**Spatial gradients are sensitive to cell constriction only when the septum provides a significant diffusion barrier.A**) Schematic of a dividing cell with oppositely localized kinase and phosphatase. *f*_*a*_is the ratio of the area of the pore at the constriction site to the cross-sectional area in the cylindrical portion of the cell. **B**) 3D mathematical modeling of the distribution of [R ∼ P] in a dividing cell with *f*_*A*_=0.3. **B**) [R ∼ P] line scan through the cell middle showing volume-weighted average density along cell length for varying constriction sizes. **D**) Schematic of 1D distribution of kinase and phosphatase activities that produce profiles mimicking those in (**C**) for *f*_*A*_ < 0.1 *μ*m, with background kinase and phosphatase localized at the middle of the cell and overlapping by *L*/10.

To investigate how the constricted morphologies during division affect gradients, we performed simulations in which we varied the fraction *f*_*a*_ of the cross-sectional area of the cell that is encompassed by the pore at the constriction site between two dividing cells (Figure 10A,B); *f*_*a*_decreases from 1 to 0 during division. We found that the gradient was not noticeably altered until the constriction was significant (*f*_*a*_≲0.3) (Figure 10C). As the amount of constriction was increased, the total amount of R ∼ P increased in the source compartment and decreased in the sink compartment, and the magnitude of the slope decreased throughout most of the cell (Figure 10C); however, there was little change in the polar ratio *η* until *f*_*a*_ was very close to zero.

We noted that the R ∼ P distribution in Figure 10C for *f*_*A*_ < 0.3 can be recapitulated in 1D simulations by adding a source and a sink adjacent to the left and right of the constriction site, respectively (Figure 10D). Across the constriction site, the magnitude of the slope is large because the length scale between the midcell-localized source and sink is short compared to the cell length. When the source and the sink are completely compartmentalized, both daughter cells have a uniform distribution of R ∼ P; in the absence of other kinases and phosphatases all response regulator molecules will be phosphorylated in the source cell and dephosphorylated in the sink cell (blue line, Figure 10C). Thus, cell division can also provide a switch-like cue, leading to one daughter cell being dominated by the phosphorylation effects of the source and the other by the dephosphorylation of the sink, providing a mechanism for asymmetric development in organisms such as *C. crescentus*.

## Discussion

Spatial asymmetry in bacterial cells is often cell-cycle regulated and highly dynamic, and a natural consequence is the production of spatial gradients. We have established that gradients produced by a localized source and sink will be robust and significant as long as the kinetics of the source and sink are on timescales faster than the typical time required to diffuse across the length of the cell (Figure 1). Thus, any localized two-component system with a phosphotransfer rate faster than ∼20/s can give rise to cellular asymmetries, which can influence the spatial regulation of downstream components of the regulatory network. These spatial effects should be considered carefully in *in vitro* assays, where the interactions between proteins may not be representative of spatial non-uniformities occurring *in vivo*. Furthermore, in *in vivo* studies, it is important to consider the effects of fluorescent protein fusion on gradient formation via changes to the diffusion constant of a response regulator; our studies provide a mathematical framework for inferring the consequences of such changes.

Just as gradient formation requires fast enzyme kinetics, in order for other biochemical processes to disrupt an existing gradient, their kinetics must be similarly fast. In our model, fluctuations in enzyme concentrations can be directly mapped to changes in the effective enzymatic rates. Therefore, as shown in Figure 1, fluctuations that increase or decrease the effective enzymatic rates will lead to a steeper or shallower gradient, respectively. For enzymatic rates higher than 100/s, the enzyme concentration would have to fluctuate significantly to perturb the gradient (Figure 1, inset). We have also demonstrated that physiological levels of synthesis, degradation and non-specific activities of other sources and sinks are all unlikely to affect existing spatial gradients, as the timescales of their actions are usually larger than tens of seconds and therefore their effects will be made uniform by diffusion (Figures 2 and 4). We have also shown that saturation of the source and/or sink will affect gradient formation only when the levels of enzyme are less than 10% of the substrate (Figure 3); saturation is therefore unlikely to impact gradient formation under conditions in which the substrate and localized enzyme levels are comparable. Molecular crowding, which can result in subdiffusive behavior of cytoplasmic proteins, could also favor the establishment of gradients by increasing the time scale for movement between the poles [26].

Our modeling also applies to pathways besides two-component systems, such as the synthesis of the cytoplasmic second messenger cyclic-di-GMP by diguanylate cyclases and degradation by phosphodiesterases. While fast kinetics are required for gradient formation, localized kinases and phosphatases can also give rise to asymmetries after cytokinesis, solely by segregating localized components. For example, cyclic-di-GMP is asymmetrically distributed in *C. crescentus* and *Pseudomonas* immediately after cell division [4]. Christen et al. suggested that the dephosphorylation activity of the unipolarly localized enzyme PleC is ultimately responsible for lowering cyclic-di-GMP levels in one of the daughter cells by inactivating the diguanylate cyclase PleD, which synthesizes cyclic-di-GMP [4]. Notably, there is no apparent asymmetry in cyclic-di-GMP levels prior to the completion of division, thus our modeling results predict that in this scenario PleD must be deactivated at a rate not significantly larger than the inverse diffusive timescale, thus requiring cell division to give rise to the asymmetry.

While cyclic-di-GMP asymmetry appears to require cell division, other bacterial pathways rely on fast kinetics of localized kinases and phosphatases to establish spatial gradients prior to division. For example, DNA replication in *C. crescentus* is regulated by a gradient of the phosphorylated form of CtrA; this gradient is formed tens of minutes prior to cytokinesis and ensures that one daughter cell is in G1 phase and the other is in S phase immediately following septation [11]. Why would a cell need spatial gradients when division can inherently provide asymmetry through compartmentalization? One possibility is timing control: establishing asymmetry early in the developmental cycle can ensure that the cell is robust to fluctuations.

We have also demonstrated that a representative sample of bacterial cell shapes and sizes can support gradient formation through oppositely localized kinase and phosphatase activities (Figures 5, 6, 7, 8 and 9) and that the variations in gradient profiles in these 3D shapes relative to a rod shape can be recapitulated in 1D simulations by varying the localization of the source and sink activities. Whereas we have found that gradients are enhanced during cell elongation due to the increase in the distance between the source and sink (Figure 5), we have also shown that increasing cellular volume in a direction orthogonal to the kinase-phosphatase axis causes flattening of the gradient (Figures 7, 8 and 9). Given that major shape changes are required to alter a gradient (Figures 5, 6, 7, 8 and 9), we expect that local perturbations to cell shape such as fluctuations in rod-shaped cell width are highly unlikely to disrupt a cellular-scale gradient.

## Conclusions

Our analysis of gradient formation encompasses a wide range regulatory mechanisms and morphologies, demonstrating the conditions under which robust spatial gradients can be realized for providing intercellular spatial cues. Our results highlight the utility of mathematical modeling in future studies of intracellular organization in bacteria, and illustrate the complex spatial patterning that can be achieved even in the absence of membrane compartmentalization.

## Methods

Unless otherwise stated, the kinase and phosphatase *σ*rates in our simulations were set to _*k*_ = *σ*_*p*_ = 100/s and the diffusion constant was *D* = 2*μ*m^2^/s. For all 1D and rod-shaped cell simulations, the cell length was *L* = 2*μ*m, unless otherwise stated. In 1D simulations each pole was assumed to occupy 0.25 *μ*m of the cell length. The reaction-diffusion equations were numerically integrated forward in time until a steady state was reached, using a time step *dt* = 0.0005 s and a spatial grid with *dx* = 0.05*μ*m.

The steady-state solutions in 3D geometries were determined using an in-house, custom-written Matlab (The Mathworks, Inc., Natick, MA, USA) software package called TURING, developed for simulating reaction-diffusion equations in complex geometries. TURING includes a graphical interface for creating finite-element grids representing biologically relevant morphologies, and a symbolic library capable of interpreting intuitively defined reaction-diffusion equations and parameters to build a model for a simulation. TURING solves the system of reaction-diffusion equations on the specified grid with a fully implicit, numerical method. The source and sink elements reside on the cell membrane, occupying the same surface area *A*_*s*_ as a pole in the case of a rod-shaped cell with radius *r*_*p*_ = 0.25 *μ*m; *A*_*s*_ = 0.39 *μ*m^2^ for both kinase and phosphatase elements. The enzymatic rates *σ*_*k*_and *σ*_*p*_ were set to 100/s for the curved cylinder geometry. These rates were appropriately scaled for the other geometries, such that the number of active kinases and phosphatases remained consistent between simulations. This corresponded to holding the products *V*_*k*_*σ*_*k*_ and *V*_*p*_*σ*_*p*_ constant for all geometries, where *V*_*k*_and *V*_*p*_ are the total volumes of the kinase and phosphatase finite elements respectively.

Cells with azimuthal symmetry were solved ignoring azimuthal diffusion. Curved cylinder, spherical, and constricting cell geometries were represented by a grid with elements whose average side lengths were 75, 10, and 25 nm, respectively.

## Competing interests

The authors declare that they have no competing interests.

## Authors’ contributions

C.T. and K.C.H. designed the study. C.T., N.R. and K.C.H. designed and performed the mathematical modeling. C.T. and K.C.H. wrote the manuscript. All authors discussed the results and commented on the manuscript. All authors read and approved the final manuscript.

## References

[B1] Lander AD Pattern, Growth, and Control Cell 2011 144 6 955 969 10.1016/j.cell.2011.03.009 21414486 PMC3128888

[B2] Shapiro L Mcadams HH Losick R Why and how bacteria localize proteins Science 2009 326 5957 1225 1228 10.1126/science.1175685 19965466 PMC7531253

[B3] Chen CH Lu Y Sin MLY Mach KE Zhang DD Gau V Liao JC Wong PK Antimicrobial susceptibility testing using high surface-to-volume ratio microchannels Anal Chem 2010 82 3 1012 1019 10.1021/ac9022764 20055494 PMC2821038

[B4] Christen M Kulasekara HD Christen B Kulasekara BR Hoffman LR Miller SI Asymmetrical distribution of the second messenger c-di-GMP upon bacterial cell division Science 2010 328 5983 1295 1297 10.1126/science.1188658 20522779 PMC3906730

[B5] Charles M Pérez M Kobil JH Goldberg MB Polar targeting of Shigella virulence factor IcsA in Enterobacteriacae and Vibrio Proc Nat Acad Sci USA 2001 98 17 9871 9876 10.1073/pnas.171310498 11481451 PMC55545

[B6] Robbins JR Monack D McCallum SJ Vegas A Pham E Goldberg MB Theriot JA The making of a gradient: IcsA (VirG) polarity in Shigella flexneri Mol Micro 2002 41 4 861 872 10.1046/j.1365-2958.2001.02552.x 11532149

[B7] Pham T Varga A Goldberg M The unipolar Shigella surface protein IcsA is targeted directly to the bacterial old pole: IcsP cleavage of IcsA occurs over the entire bacterial surface Mol Micro 1999 32 2 367 377 10.1046/j.1365-2958.1999.01356.x 10231492

[B8] Lipkow K Andrews SS Bray D Simulated diffusion of phosphorylated CheY through the cytoplasm of Escherichia coli J Bacteriol 2005 187 45 53 10.1128/JB.187.1.45-53.2005 15601687 PMC538814

[B9] Vaknin AA Berg HCH Single-cell FRET imaging of phosphatase activity in the Escherichia coli chemotaxis system Proc Nat Acad Sci USA 2004 101 49 17072 17077 10.1073/pnas.0407812101 15569922 PMC535373

[B10] Werner JN Chen EY Guberman JM Zippilli AR Irgon JJ Gitai Z Quantitative genome-scale analysis of protein localization in an asymmetric bacterium Proc Nat Acad Sci USA 2009 106 19 7858 7863 10.1073/pnas.0901781106 19416866 PMC2671984

[B11] Chen YE Tropini C Jonas K Tsokos CG Huang KC Laub MT Spatial gradient of protein phosphorylation underlies replicative asymmetry in a bacterium Proc Nat Acad Sci USA 2011 108 3 1052 1057 10.1073/pnas.1015397108 21191097 PMC3024676

[B12] McGrath PT Viollier P Mcadams HH Setting the pace: mechanisms tying Caulobacter cell-cycle progression to macroscopic cellular events Curr Opin Microbiol 2004 7 2 192 197 10.1016/j.mib.2004.02.012 15063858

[B13] Brown GC Kholodenko BN Spatial gradients of cellular phospho-proteins FEBS Lett 1999 457 3 452 454 10.1016/S0014-5793(99)01058-3 10471827

[B14] Kholodenko BN Brown GC Hoek JB Diffusion control of protein phosphorylation in signal transduction pathways Biochem J 2000 350 Pt 3 901 907 10970807 PMC1221325

[B15] Lipkow K Odde DJ Model for Protein Concentration Gradients in the Cytoplasm Cell Mol Bioeng 2008 1 84 92 10.1007/s12195-008-0008-8 21152415 PMC2996619

[B16] Endres RG Wingreen NS Accuracy of direct gradient sensing by single cells Proc Nat Acad Sci USA 2008 105 41 15749 15754 10.1073/pnas.0804688105 18843108 PMC2572938

[B17] Elowitz MB Surette MG Wolf PE Stock JB Leibler S Protein mobility in the cytoplasm of Escherichia coli J Bacteriol 1999 181 197 203 9864330 10.1128/jb.181.1.197-203.1999 PMC103549

[B18] Meacci G Ries J Fischer-Friedrich E Kahya N Schwille P Kruse K Mobility of Min-proteins in Escherichia coli measured by fluorescence correlation spectroscopy Phys Bio 2006 3 4 255 263 10.1088/1478-3975/3/4/003 17200601

[B19] Mayover TL Halkides CJ Stewart RC Kinetic characterization of CheY phosphorylation reactions: comparison of P-CheA and small-molecule phosphodonors Biochemistry 1999 38 8 2259 2271 10.1021/bi981707p 10029518

[B20] Nath K Koch AL Protein degradation in Escherichia coli. II. Strain differences in the degradation of protein and nucleic acid resulting from starvation J Biol Chem 1971 246 22 6956 6967 4942328

[B21] Young R Bremer H Polypeptide-chain-elongation rate in Escherichia coli B/r as a function of growth rate Biochem J 1976 160 2 185 194 795428 10.1042/bj1600185 PMC1164221

[B22] Skerker JM Laub MT Cell-cycle progression and the generation of asymmetry in Caulobacter crescentus Nat Rev Microbiol 2004 2 4 325 337 10.1038/nrmicro864 15031731

[B23] Young KD The selective value of bacterial shape Microbiol Mol Biol R 2006 70 3 660 703 10.1128/MMBR.00001-06 PMC1594593 16959965

[B24] Takeuchi S DiLuzio WR Weibel DB Whitesides GM Controlling the shape of filamentous cells of Escherichia coli Nano Lett 2005 5 9 1819 1823 10.1021/nl0507360 16159230 PMC2519610

[B25] Männik J Driessen R Galajda P Keymer JE Dekker C Bacterial growth and motility in sub-micron constrictions Proc Nat Acad Sci USA 2009 106 35 14861 14866 10.1073/pnas.0907542106 19706420 PMC2729279

[B26] Banks DS Fradin C Anomalous diffusion of proteins due to molecular crowding Biophys J 2005 89 5 2960 2971 10.1529/biophysj.104.051078 16113107 PMC1366794

